# Complete genome sequences of the *Paenibacillus kyungheensis* KACC 18744^T^, *Sphingomonas naphthae* KACC 18716^T^, and *Novosphingobium humi* KACC 19094^T^

**DOI:** 10.1128/mra.00753-24

**Published:** 2025-01-27

**Authors:** Hyorim Choi, Seunghwan Kim, Miyoung Won, Yunhee Choi, Yonghoon Lee, Yiseul Kim, Jun Heo

**Affiliations:** 1 Agricultural Microbiology Division, National Institute of Agricultural Sciences, Rural Development Administration, Wanju-gun, South Korea; 2 Division of Biotechnology, Jeonbuk National University, Iksan-si, South Korea; 3 Subtropical Livestock Research Institute, National Institute of Animal Sciences, Rural Development Administration, Jeju-si, South Korea; 4 Division of Life Sciences, Jeonbuk National University, Jeonju-si, South Korea; Indiana University, Bloomington, Bloomington, Indiana, USA

**Keywords:** *Paenibacillus*, *Novosphingobium*, *Sphingomonas*, genomes, type strain

## Abstract

We report the whole genome sequences of *Paenibacillus kyungheensis* KACC 18744^T^, *Sphingomonas naphthae* KACC 18716^T^, and *Novosphingobium humi* KACC 19094^T^, to investigate the genomic diversity of bacterial type strains distributed in Korea.

## ANNOUNCEMENT

The genomic information of type strains plays a crucial role in bacterial phylogenetics, functional gene analysis, and comparative genomic analyses. Despite its importance, genome analysis was not mandatory for the description of novel bacterial species prior to 2018 (1). Many type strains reported before this time still lack sufficient genomic data. To address this gap, we conducted genome sequencing of type strains preserved at the Korean Agricultural Culture Collection (KACC). Specifically, our study focused on three type strains (*Paenibacillus kyungheensis* KACC 18744^T^, *Sphingomonas naphthae* KACC 18716^T^, and *Novosphingobium humi* KACC 19094^T^) isolated and reported in Korea between 2015 and 2017 (2–4), but genomic data for these strains remained unavailable until now.

These strains were cultured on Reasoner’s 2A medium (BD Difco, NJ, USA) with pH 6.0 at 28°C for 3 days under aerobic condition. According to the manufacturer’s protocol, the Qiagen MagAttract HMW DNA kit (Qiagen, Hilden, Germany) was used for genomic DNA extraction. The DNA products generated from the previous procedure were used for genome sequence analysis on an Illumina MiSeq (Illumina, CA, USA) and PacBio Sequel IIe (Pacific Biosciences, CA, USA). Genomic DNA libraries were created using the TruSeq Nano DNA High Throughput Library Prep kit (Illumina, CA, USA). A total of 10 µL libraries were prepared as 7–12kb size templates for the PacBio SMRTbell prep kit 3.0. Then, they were analyzed using the Sequel II Bind Kit 3.2 and Int Ctrl 3.2. Sequencing was carried out using Sequel II Sequencing Kit 2.0 and SMRT cell 8M trays. HIFI reads were obtained from the PacBio Sequel IIe system and assembled using the microbial assembly application in SMRT link 11.0.0.146107 software with default parameters, based on the Hierarchical Genome Assembly Process (5). HIFI reads were generated with quality value 20 or 99% predicted accuracy.

The Illumina raw reads of which 90% of the bases had a phred score of 30 or higher were filtered, and adapter trimming was performed using Trimmomatic 0.38 (6). Then, the assembly, initially constructed with long-read data, was revised through Pilon v1.21 to correct errors and enhance precision (7). Successfully circularized were confirmed by microbial genome analysis application in the SMRTlink 11.0.0.146107 (8), and completeness were checked by BUSCO v5 (9). Gene prediction and annotation were conducted by the NCBI Prokaryotic Genome Annotation Pipeline v6.7 (PGAP) (10). All tools were executed with default parameters unless stated otherwise.

The genome of *P. kyungheensis* KACC 18744^T^ consists of a single circular chromosome (5,260,882 bp with GC content of 39.5%). The genome of *S. naphthae* KACC 18716^T^ has a single circular chromosome (4,310,851 bp with GC content of 67.5%) and four other contigs. The genome of *N. humi* KACC 19094^T^ has a single circular chromosome (4,890,578 bp with GC content of 63.5%) and three other contigs. Additional details and annotation results for the three genomes are shown in Table 1.

**TABLE 1:** Sequencing, assembly, and annotation results for the sequenced strains

Strain	KACC 18744^T^	KACC 18716^T^	KACC 19094^T^
Species	*Paenibacillus kyungheensis*	*Sphingomonas naphthae*	*Novosphingobium humi*
BioProject accession no.	PRJNA992936	PRJNA930972	PRJNA930975
BioSample accession no.	SAMN36377341	SAMN33038042	SAMN33039045
GenBank assembly accession no.	GCF_028606985	GCF_028607085	GCF_028607105
GenBank accession no.	CP117416	CP117411-CP117415	CP117417-CP117420
SRA accession no.			
Illumina	SRR25238406	SRR23952290	SRR24694047
Pacbio	SRR25238405	SRR23952291	SRR24694046
HiFi reads			
Total length (bp)	1,227,207,177	1,714,875,056	1,475,901,199
Total no. of reads	136,392	190,527	171,360
*N*_50_ (bp)	9,721	9,666	9,271
Mean quality	Q35	Q32	Q33
Illumina			
Total length (bp)	3,355,346,840	3,219,202,522	3,158,184,630
Paired length (bp)	2,623,250,643	2,342,773,538	2,402,495,342
Filtered no. of reads	17,380,330	15,521,426	15,915,742
Q20 (%)	99.36	99.37	99.35
*De novo* assembly			
No. of contigs	1 (circular)	5 (circular)	4 (circular)
Total length (bp)	5,258,865	4,309,746	4,890,308
Corrected total length	5,260,882	4,310,851	4,890,578
G+C content (%)	39.3	67.3	63.6
Sequencing depth	233.0×	397.2×	301.4×
BUCO completion (%)	99.19	100	99.19
Chromosome length (bp)	5,260,882	3,919,827	3,439,207
Annotation results			
No. of genes	4,586	4,199	4,435
No. of CDS	4,480	4,143	4,368
No. of RNA genes	106	56	67

## Data Availability

The genomes are available in GenBank under the accession numbers shown in Table 1.
